# Integrating Multivariate Ordination and Machine Learning to Disentangle the Environmental Drivers of Xylem Sap Redox Metabolism in Trees

**DOI:** 10.3390/plants15172549

**Published:** 2026-08-22

**Authors:** Rıfat Kurt, Zeynep Eda Özan

**Affiliations:** Department of Forest Industrial Engineering, Faculty of Forestry, Bartin University, 74100 Bartin, Türkiye; z.edaozan@hotmail.com

**Keywords:** antioxidant defense, machine learning, meteorological drivers, multivariate statistics, oxidative stress, tree species, xylem sap

## Abstract

Xylem sap is increasingly recognized as a dynamic biological matrix reflecting whole-plant physiological status, but its biochemical variation under field conditions remains insufficiently characterized. We investigated oxidative stress markers, osmolytes, antioxidant enzymes, and redox-related enzymes in xylem sap from three focal tree individuals representing *Fraxinus excelsior*, *Populus nigra*, and *Pinus sylvestris*. Sap was collected by passive stem tapping using a custom-built apparatus, and biochemical patterns were evaluated using multivariate statistical and machine-learning approaches. The three focal trees showed distinct biochemical profiles within the present dataset. The focal *P. nigra* individual was associated with relatively higher antioxidant enzyme activities, whereas the focal *F. excelsior* and *P. sylvestris* individuals were more closely associated with oxidative-damage and metabolic-adjustment traits. Precipitation and wind direction were retained as the main meteorological variables associated with biochemical variation, with wind direction interpreted as an atmospheric correlate rather than a direct physiological driver. Exploratory machine-learning analyses highlighted catalase and selected meteorological variables as influential predictors. Overall, the findings support the potential of xylem sap for integrative ecophysiological monitoring while emphasizing the exploratory nature of patterns derived from repeated measurements of three focal trees.

## 1. Introduction

Plants are exposed to substantial environmental variability, including fluctuations in temperature, precipitation, relative humidity, and solar radiation, and ongoing climate change is expected to intensify many of these stressors [1]. Understanding how such variability influences plant physiological and biochemical responses is therefore central to plant ecophysiology and stress biology.

Environmental stress can disrupt cellular redox homeostasis and increase the production of reactive oxygen species (ROS). Although ROS function as important signaling molecules, excessive accumulation can damage cellular components [2,3]. Plants counter such imbalances through integrated antioxidant defense mechanisms [3,4]. Stress-related metabolites and osmotic-adjustment responses also contribute to physiological adaptation under adverse environmental conditions. Collectively, these biochemical responses provide important indicators for determining the physiological status and stress levels of plants under changing environmental conditions.

Xylem sap functions not only as a medium for water and mineral transport but also as a dynamic component of plant-wide physiological and biochemical signaling. It contains metabolites, proteins, peptides, hormones, and defense-related compounds that can reflect both environmental conditions and internal physiological processes [5,6,7,8,9,10]. Evidence that xylem-borne compounds participate in long-distance stress signaling and systemic defense further supports the potential of xylem sap as an integrative matrix for ecophysiological monitoring [6,8,10,11]. Accordingly, biochemical characterization of xylem sap may provide valuable information on plant physiological status and responses to environmental variability.

Despite growing interest in xylem sap as a source of physiological information, previous studies have largely focused on its molecular composition, proteomic characterization, or specific signaling responses. Comparatively less attention has been given to the simultaneous variation of oxidative-stress markers, osmolytes, antioxidant enzymes, and redox-related enzymes in xylem sap under field conditions. Moreover, studies integrating repeated xylem-sap measurements with seasonal and meteorological information within a common multivariate analytical framework remain limited. Addressing this gap may provide a more integrated view of how xylem-sap biochemical profiles vary in association with temporal and environmental conditions.

Biomarkers such as malondialdehyde (MDA), hydrogen peroxide (H_2_O_2_), and antioxidant enzymes like SOD, CAT, and APX are widely used to evaluate oxidative stress and antioxidant defense networks in plants, and these parameters have been shown to be sensitive to environmental stress in numerous studies [2,3,12,13,14,15].

In field-based ecophysiological studies, the multidimensional effects of species identity alongside climatic, seasonal, and edaphic variability on plant functional traits are resolved through the integration of ordination methods such as Principal Component Analysis (PCA) [16] and Redundancy Analysis (RDA) [17,18] along with permutational multivariate analysis of variance (PERMANOVA) to test for differences between groups [19,20] and machine learning techniques like Random Forest to allow for variable importance ranking [21,22,23,24]. These multivariate approaches highlight the decisive role of climatic and seasonal drivers [25,26] alongside species-specific physiological responses in determining plant performance [27]. However, the vast majority of these studies focus on leaves, needles, or other plant tissues, whereas ecophysiological evaluations based on xylem sap remain limited.

The aim of this study was to evaluate variation in oxidative stress markers, osmolytes, antioxidant enzymes, and redox-related enzymes in xylem sap obtained from the three focal tree individuals representing *F. excelsior*, *P. nigra*, and *P. sylvestris*. The associations of meteorological conditions with these biochemical patterns were further examined using multivariate statistical and machine-learning approaches together with the composite Oxidative Stress Index (OSI) and Antioxidant Defense Index (ADI). Accordingly, the present study combines repeated xylem-sap biochemical measurements with meteorological records and complementary multivariate and machine-learning approaches to examine patterns associated with focal tree identity, sampling period, and environmental variability. Rather than testing generalizable species effects, the study provides an exploratory framework for integrating multiple biochemical indicators within a field-based xylem-sap monitoring context. This approach is intended to generate hypotheses regarding the environmental and temporal structuring of xylem-sap redox metabolism and to provide a methodological basis for future studies with broader biological replication.

## 2. Results

### 2.1. Variation in Oxidative Stress Markers, Osmolytes and Antioxidant Enzymes

Xylem-sap biochemical traits showed marked variation across observations from the three focal trees and sampling seasons (Table S1). The highest H_2_O_2_ value (77.74 mmol kg^−1^) was recorded in the *P. nigra*-spring group, followed by *P. sylvestris*-summer samples (76.34 mmol kg^−1^). For MDA, a marker of lipid peroxidation, the highest value (99.82 mmol kg^−1^) was measured in a *F. excelsior*-autumn sample; similarly high MDA levels were observed in winter samples of *F. excelsior* (96.96 mmol kg^−1^ and 94.71 mmol kg^−1^). These results indicate that peaks in ROS accumulation (H_2_O_2_) and the resulting oxidative damage (MDA) do not always coincide in the same focal tree–season combinations, reflecting a heterogeneous expression of stress across groups.

Responses of the antioxidant system also showed high variability. In particular, POD and SOD activities reached very high levels in some *P. nigra* samples (POD: 31,043.44 U g^−1^; SOD: 2231.49 U g^−1^). GR, which plays a critical role in redox balance, reached its maximum in *F. excelsior*-autumn samples (858.12 U g^−1^), while 6PGD activity, associated with NADPH production, was highest in the *P. sylvestris*-spring group (854.78 U g^−1^). Overall, biochemical parameters did not change uniformly but shifted across a broad metabolic network across the focal trees and sampling seasons.

### 2.2. Relationships Among Meteorological Variables and Biochemical Parameters

**Figure 1:**
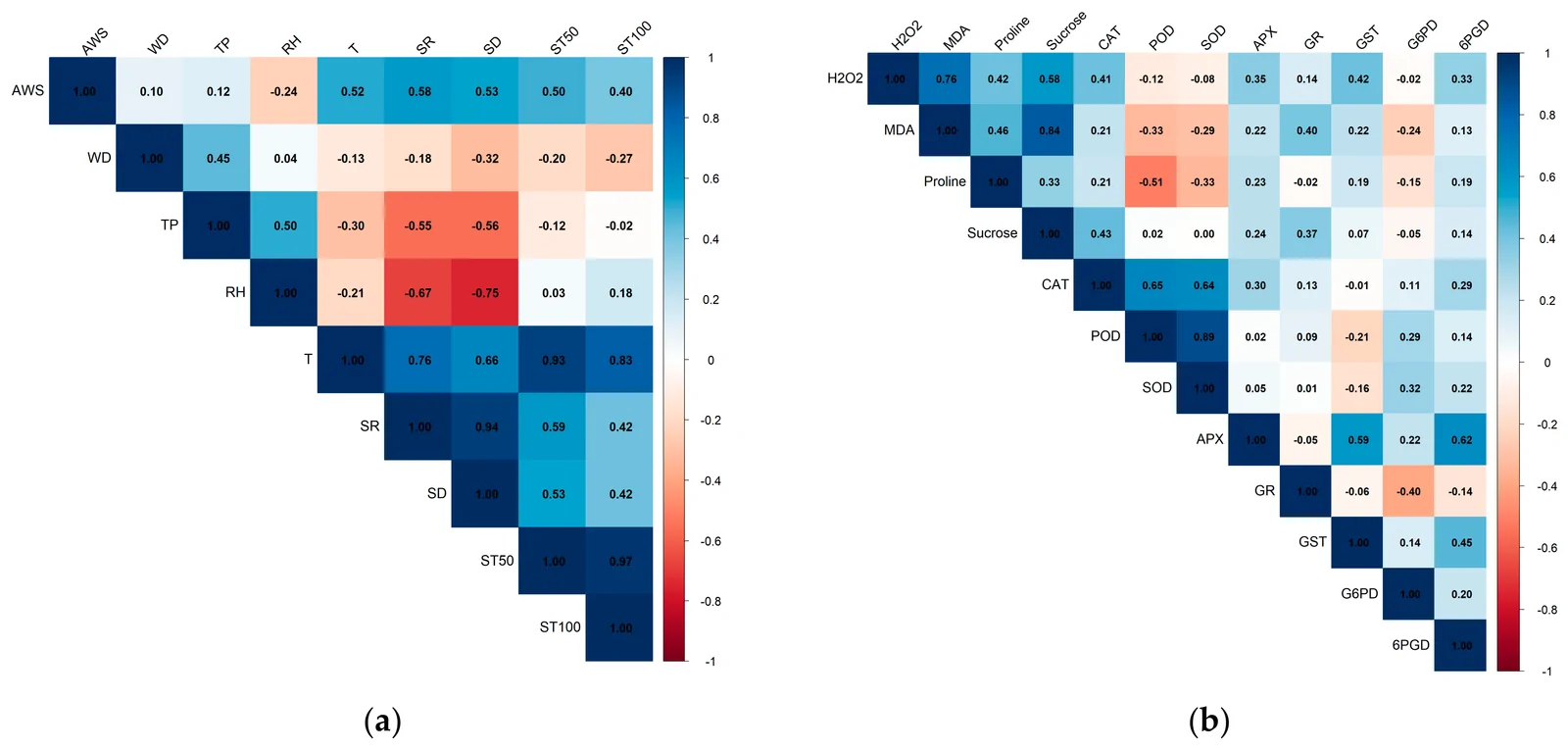
Pearson correlation matrices among meteorological variables and biochemical traits. (**a**) Meteorological variables. (**b**) Oxidative stress markers, osmolytes, and antioxidant enzymes.

Analysis of relationships among meteorological variables revealed strong and significant correlation structures, indicating multicollinearity in the dataset (Figure 1a). The most notable positive correlations were found between air temperature and soil temperatures (ST50: r = 0.93; ST100: r = 0.83). There was also a very high linear relationship between SR and SD (r = 0.94), indicating that thermal and radiative variables co-vary. Conversely, relative humidity showed strong negative correlations with both solar radiation and sunshine duration, a common meteorological pattern. These high correlation coefficients highlight the need to reduce the number of predictors in subsequent multivariate models, for which VIF analysis is appropriate.

The pooled correlation matrix showed several notable associations among biochemical variables (Figure 1b). A strong positive correlation was observed between H_2_O_2_ and MDA (r = 0.76), while MDA was also strongly positively correlated with sucrose (r = 0.84). Among antioxidant enzymes, POD and SOD showed a strong positive correlation (r = 0.89), and CAT was positively correlated with both POD (r = 0.65) and SOD (r = 0.64). Among redox-related enzymes, APX showed positive associations with 6PGD (r = 0.62) and GST (r = 0.59). In contrast, proline was negatively correlated with POD (r = −0.51). Because pooled correlations may partly reflect differences among focal tree identities or seasonal sampling structure, the two strong relationships specifically examined in the adjusted analysis were reassessed after controlling for focal tree identity and season. The H_2_O_2_–MDA association remained strong and increased slightly after adjustment (adjusted r = 0.85, *p* < 0.001; Figure S1), whereas the POD–SOD association also remained strong despite a modest reduction in magnitude (adjusted r = 0.83, *p* < 0.001; Figure S1). These results indicate that these two associations were not solely attributable to differences among the three focal trees or to seasonal grouping within the present dataset. The remaining pooled biochemical correlations were interpreted descriptively, with consideration of the grouped sampling structure.

### 2.3. Principal Component Analysis of Biochemical Responses

**Figure 2:**
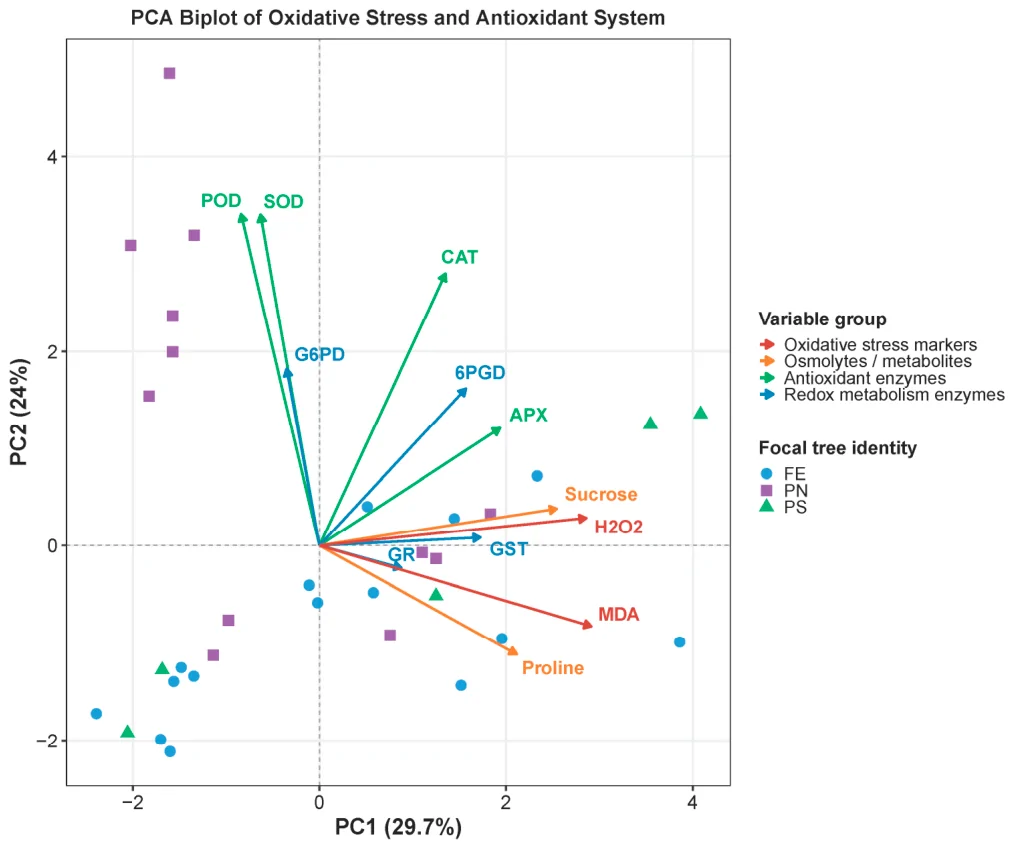
PCA biplot showing multivariate patterns of oxidative stress and antioxidant responses among observations from the three focal trees.

PCA used to identify major patterns of variation in the biochemical defense and stress profiles revealed that the first two components explained 53.67% of the total variance (PC1: 29.69%; PC2: 23.98%) (Figure 2). PC1 was most strongly loaded by oxidative stress markers (MDA, H_2_O_2_) and osmolytes (sucrose, proline) and broadly represents “stress intensity”. PC2 was dominated by antioxidant enzyme activities (POD, SOD, CAT) and reflects the plant’s “enzymatic defense capacity”. In the biplot, *P. nigra* samples generally group with high POD and SOD activities, whereas several *F. excelsior* and *P. sylvestris* samples cluster on the positive side of PC1, indicating higher oxidative stress and sugar content. The proximity of variable vectors (e.g., MDA–proline–sucrose and POD–SOD–CAT) indicates similar multivariate association patterns among variables belonging to related functional groups within the present dataset.

### 2.4. Biochemical Differentiation Among the Three Focal Trees

An exploratory PERMANOVA of the repeated observations returned F = 2.37, *p* = 0.008, and R^2^ = 0.14, indicating multivariate differentiation among observations associated with the three focal tree individuals within the present dataset (Table 1). However, because each species was represented by only one repeatedly sampled individual, this separation cannot be interpreted as an independent or generalizable species-level effect. The remaining variation reflects within-tree temporal variability and other environmental or unmeasured factors.

### 2.5. Redundancy Analysis Based on Focal Tree Identity

**Figure 3:**
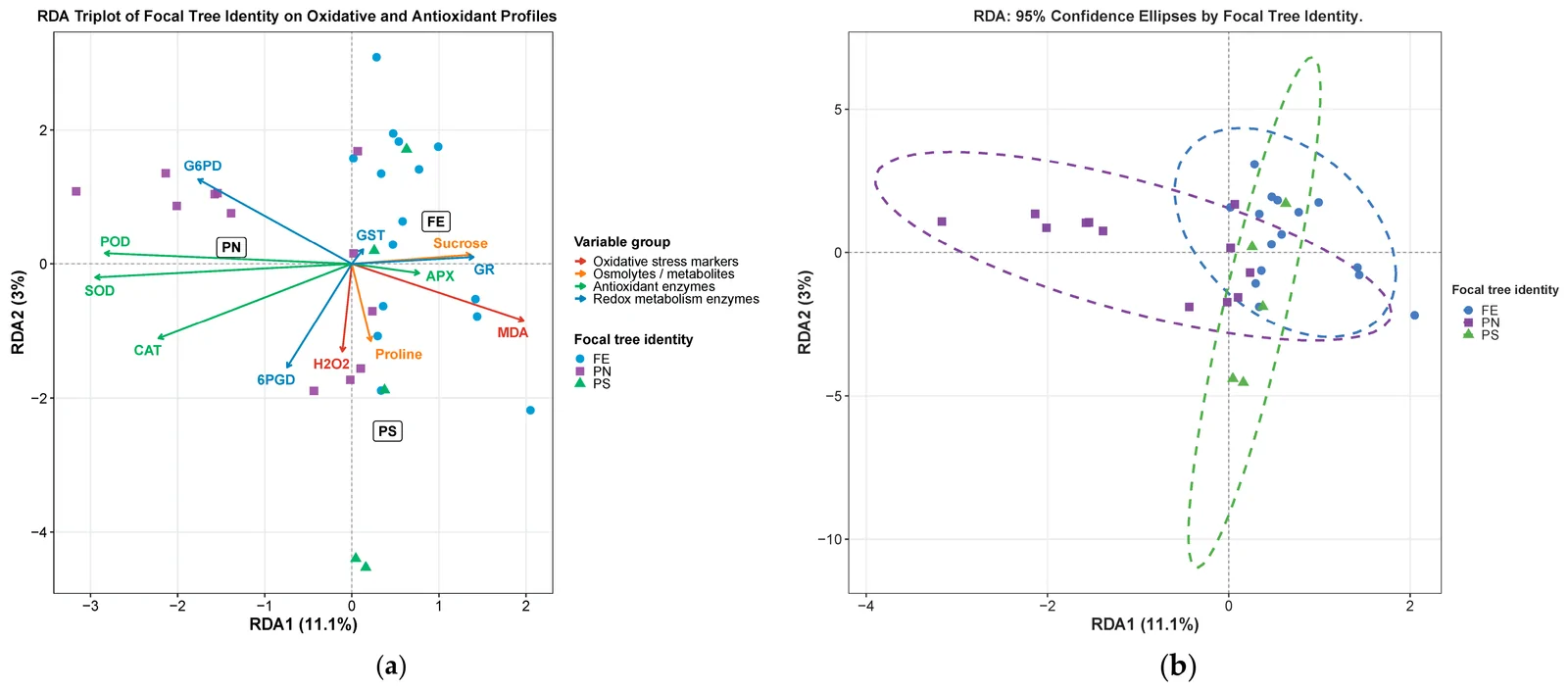
RDA of biochemical traits by focal tree identity: (**a**) RDA triplot showing differentiation among observations from the three focal trees; (**b**) 95% confidence ellipses by focal tree identity.

An exploratory RDA using focal tree identity as a descriptive grouping factor accounted for 14.1% of the total biochemical variation and was statistically significant (*p* < 0.01). This result indicates multivariate differentiation among repeated observations associated with the three focal trees within the present dataset rather than a generalizable species-level effect. In Figure 3a, observations from the focal *P. nigra* individual are positioned predominantly on the negative side of RDA1 and are associated with relatively higher POD, SOD, CAT, and G6PD activities. Observations from the focal *F. excelsior* individual are positioned mainly on the positive side of RDA1 and are associated primarily with MDA, sucrose, and GR. The focal *P. sylvestris* observations show a broader distribution in the ordination space, particularly along RDA2. The 95% confidence ellipses in Figure 3b further illustrate differences in the location and dispersion of observations among the three focal trees. Overall, the ordination supports distinct within-dataset biochemical structuring among the three focal trees, with observations from the focal *P. nigra* individual more closely associated with antioxidant enzyme activities and observations from the focal *F. excelsior* and *P. sylvestris* individuals more closely associated with oxidative-damage and metabolic-adjustment traits. These patterns should be interpreted as characteristics of the monitored focal individuals rather than as evidence of generalizable species-specific defense strategies.

### 2.6. Effects of Meteorological Variables on Biochemical Responses

VIF analysis was applied to assess multicollinearity among the meteorological predictors. Initial VIFs indicated high collinearity for several variables. Using iterative VIF elimination, ST50, SD and T were removed from the model in that order, yielding a final candidate set of AWS, WD, TP, RH, SR and ST100 (Table 2). A subsequent forward-selection procedure based on adjusted R^2^ showed that only TP and WD explained a significant portion of the biochemical variation. Accordingly, TP and WD were used as explanatory variables in the constrained ordination analyses that follow (Table 2).

The RDA (Meteorology) model built using TP and WD explained 21.5% of the total variation in biochemical parameters and was highly significant. Axis-wise permutation tests showed that most of the explained variation was concentrated on RDA1 (17.0%), while RDA2 contributed a smaller portion (4.5%) (RDA1: *p* = 0.001; RDA2: *p* = 0.10). Thus, the meteorological signal structuring biochemical responses appears to be aligned primarily along a single dominant gradient.

**Figure 4:**
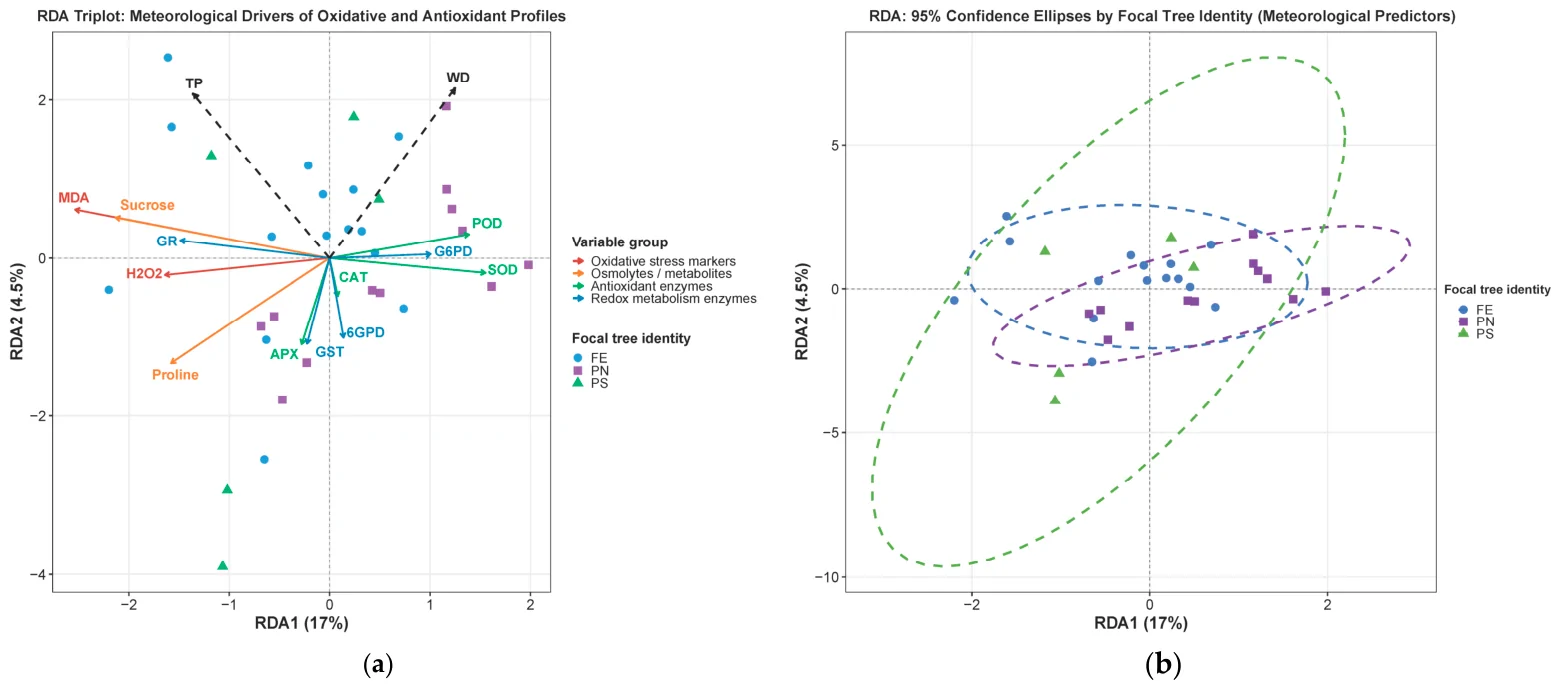
RDA of biochemical traits with meteorological drivers: (**a**) Triplot showing relationships between selected drivers (TP, WD) and biochemical variables; (**b**) 95% confidence ellipses by focal tree identity.

Figure 4a shows that TP and WD vectors shape biochemical responses in distinct directions. The WD vector extends along the positive direction of RDA1 and aligns with antioxidant enzymes such as POD, SOD, and G6PD. This pattern indicates a statistical association between wind direction and variation in antioxidant enzyme activities, particularly POD, SOD, and G6PD, without implying a direct causal effect of wind direction on enzymatic activation. The TP vector extends toward the negative direction of RDA1 and the positive direction of RDA2, displaying a closer relationship with MDA, sucrose, and GR, suggesting that precipitation covaries with oxidative-stress and metabolic-adjustment traits in the present dataset. Proline, GST, APX, and 6PGD are positioned in the lower region of the ordination, representing different stress response pathways.

Figure 4b illustrates the distribution of observations from the three focal trees along the meteorological RDA gradients. Although the ellipses partially overlap, their positions and dispersion differ within the present dataset. Observations from the focal *P. nigra* individual are concentrated mainly on the positive side of RDA1, closer to the antioxidant-enzyme region aligned with WD, whereas observations from the focal *F. excelsior* individual occupy a more central distribution. The focal *P. sylvestris* observations show broader dispersion along both RDA1 and RDA2. Given the one-individual-per-species design and unequal seasonal representation, these differences should be interpreted as within-dataset patterns among the monitored focal trees rather than as evidence of species-specific physiological plasticity or adaptive strategies.

### 2.7. Exploratory Partitioning of Variation Associated with Focal Tree Identity, Season, and Meteorological Factors

**Figure 5:**
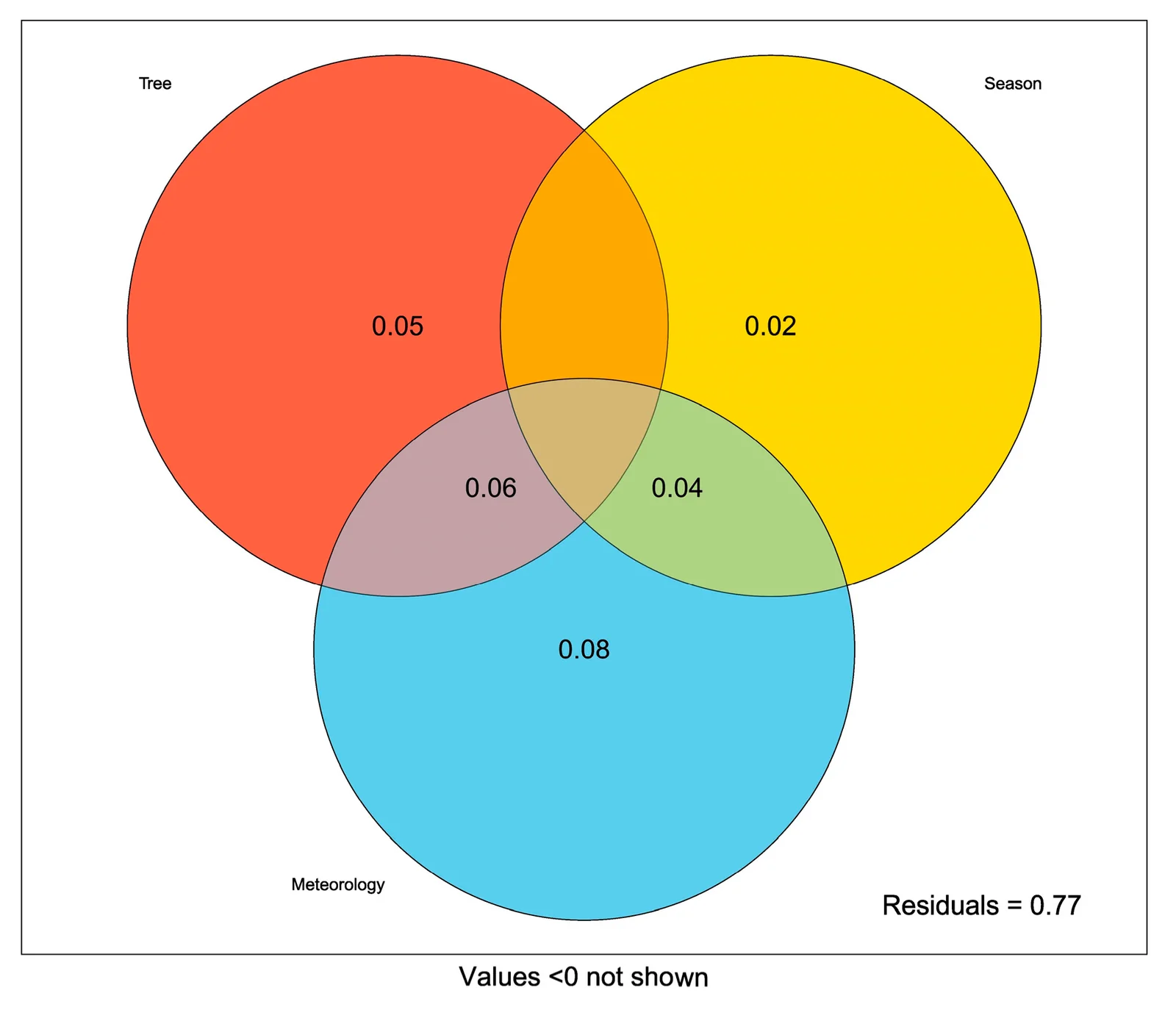
Explained variance fractions from variation partitioning analysis.

Exploratory variation partitioning was used to summarize the variation associated with focal tree identity, sampling season, and the selected meteorological variables. Because seasonal coverage was incomplete and unbalanced among the three focal trees, the fractions attributed to focal tree identity and season should not be interpreted as fully independent biological effects. The unique and shared fractions are therefore presented descriptively as within-dataset patterns (Figure 5).

The full model (Focal Tree Identity + Season + Meteorology) explained 23.1% of the total variation in biochemical parameters, while the remaining 76.9% represented unexplained variation potentially associated with unmeasured environmental, microhabitat, physiological, and other factors. Among the adjusted fractions, the largest unique fraction was associated with meteorological variables (8.4%), followed by focal tree identity (4.8%) and season (2.4%). Shared fractions included meteorology with focal tree identity (6.5%) and meteorology with season (4.0%). Because focal tree identity and season were incompletely crossed and species identity was fully confounded with individual-tree identity, these fractions are interpreted descriptively rather than as independent causal effects. Negative adjusted fractions were not assigned a biological interpretation because such values can arise from shared explanatory structure and adjustment in variation-partitioning analyses. Overall, meteorological variables accounted for the largest unique fraction of explained biochemical variation within the present dataset (Figure 5).

### 2.8. Random Forest Identification of Key Drivers

#### 2.8.1. Exploratory Classification by Focal Tree Identity

**Figure 6:**
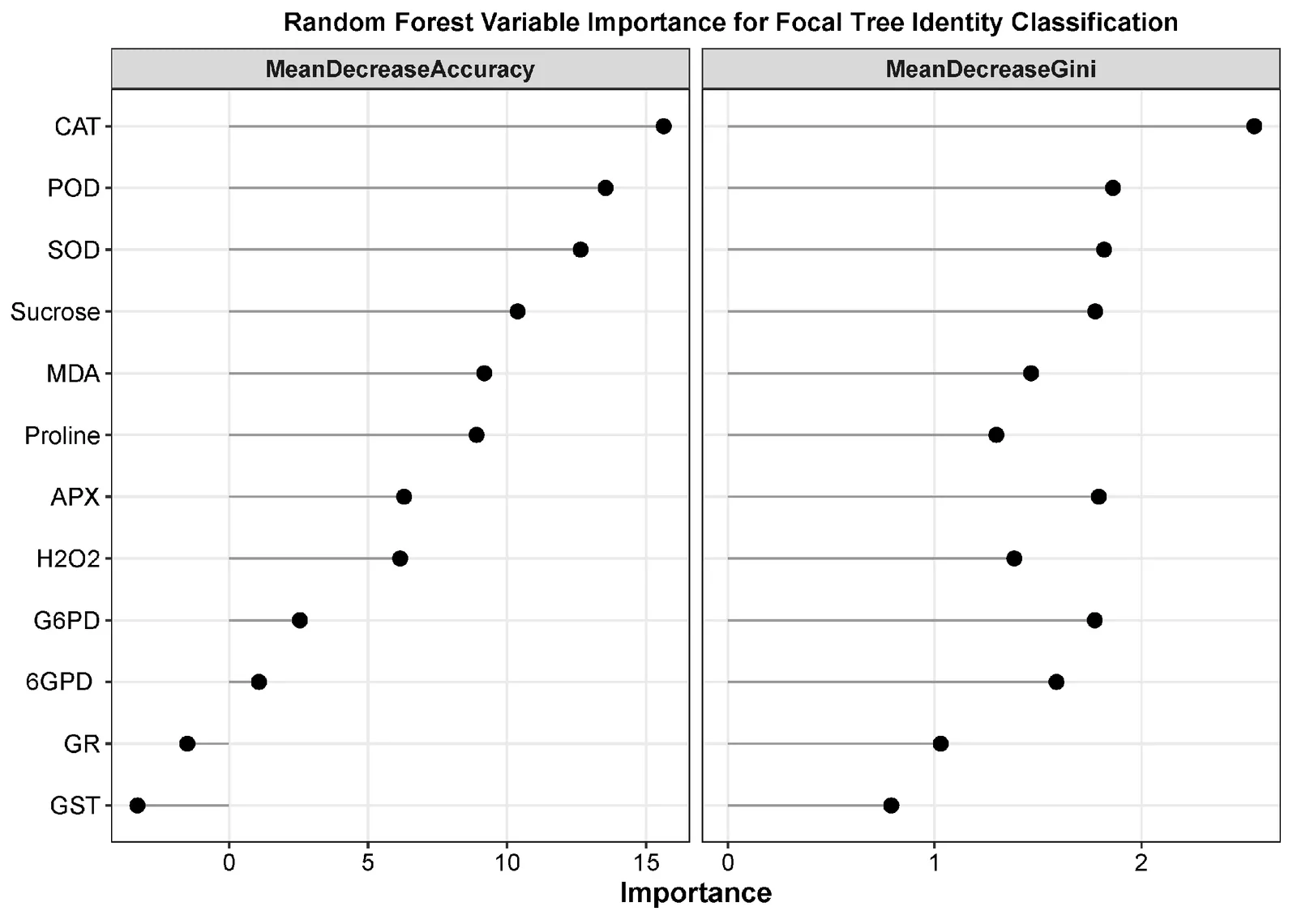
Random Forest variable importance for exploratory classification by focal tree identity based on biochemical traits (Mean Decrease Accuracy and Mean Decrease Gini).

An exploratory Random Forest classification was used to assess which biochemical variables contributed most strongly to the classification of observations by focal tree identity. According to both Mean Decrease Accuracy and Mean Decrease Gini, CAT showed the highest variable importance, followed by POD and SOD (Figure 6). Sucrose and MDA also contributed to classification, whereas GST and GR showed relatively lower importance. These rankings identify the biochemical variables contributing most strongly to differentiation among observations from the three focal trees within the present dataset and should not be interpreted as validated species-specific biochemical fingerprints.

#### 2.8.2. Drivers of Oxidative Stress Index (OSI) and Antioxidant Defense Index (ADI)

**Figure 7:**
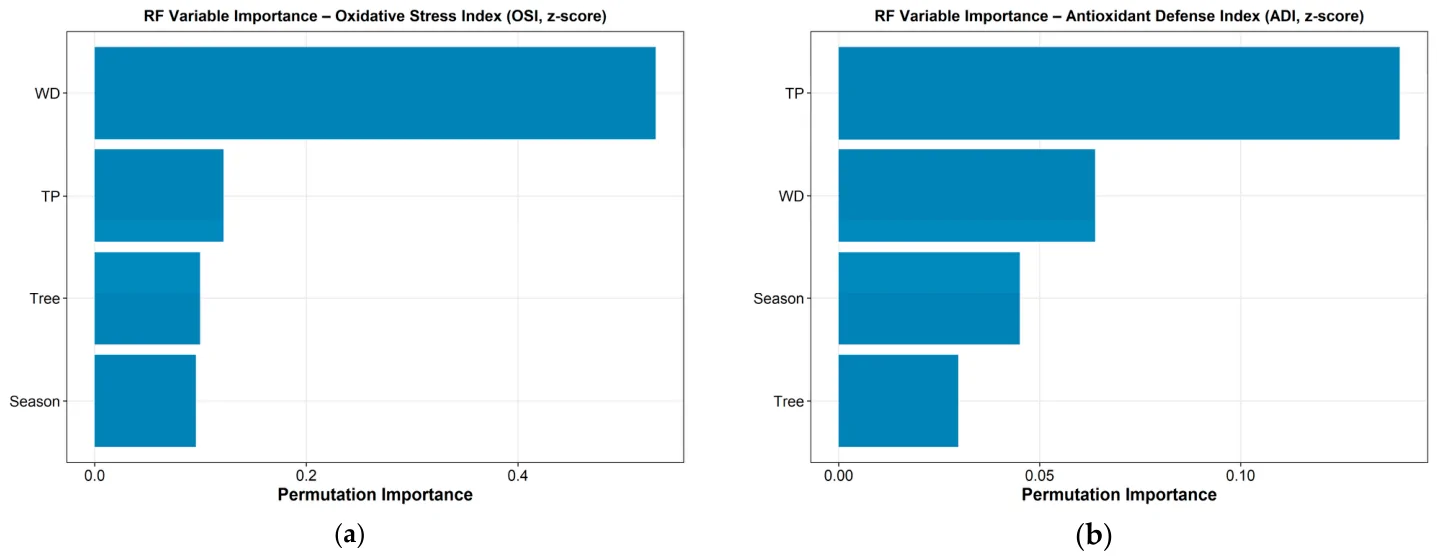
Random Forest variable importance for stress indices: (**a**) Oxidative Stress Index (OSI, z-score) and (**b**) Antioxidant Defense Index (ADI, z-score). Variable importance is based on permutation importance.

Random Forest regression was used exploratorily to evaluate predictors associated with OSI and ADI derived from the biochemical data (Figure 7). For OSI, WD showed the highest variable importance, followed by TP and focal tree identity. For ADI, TP ranked first, followed by WD and season. These rankings indicate that the two composite indices were associated with different predictor structures within the present dataset. The relatively higher importance of focal tree identity in the OSI model should be interpreted as variation associated with the three monitored individuals rather than as an independent species effect. Overall, the results suggest that oxidative-stress and antioxidant-defense indices may be associated with different combinations of meteorological, temporal, and focal-tree-related variation.

### 2.9. Hierarchical Clustering of Biochemical Traits

**Figure 8:**
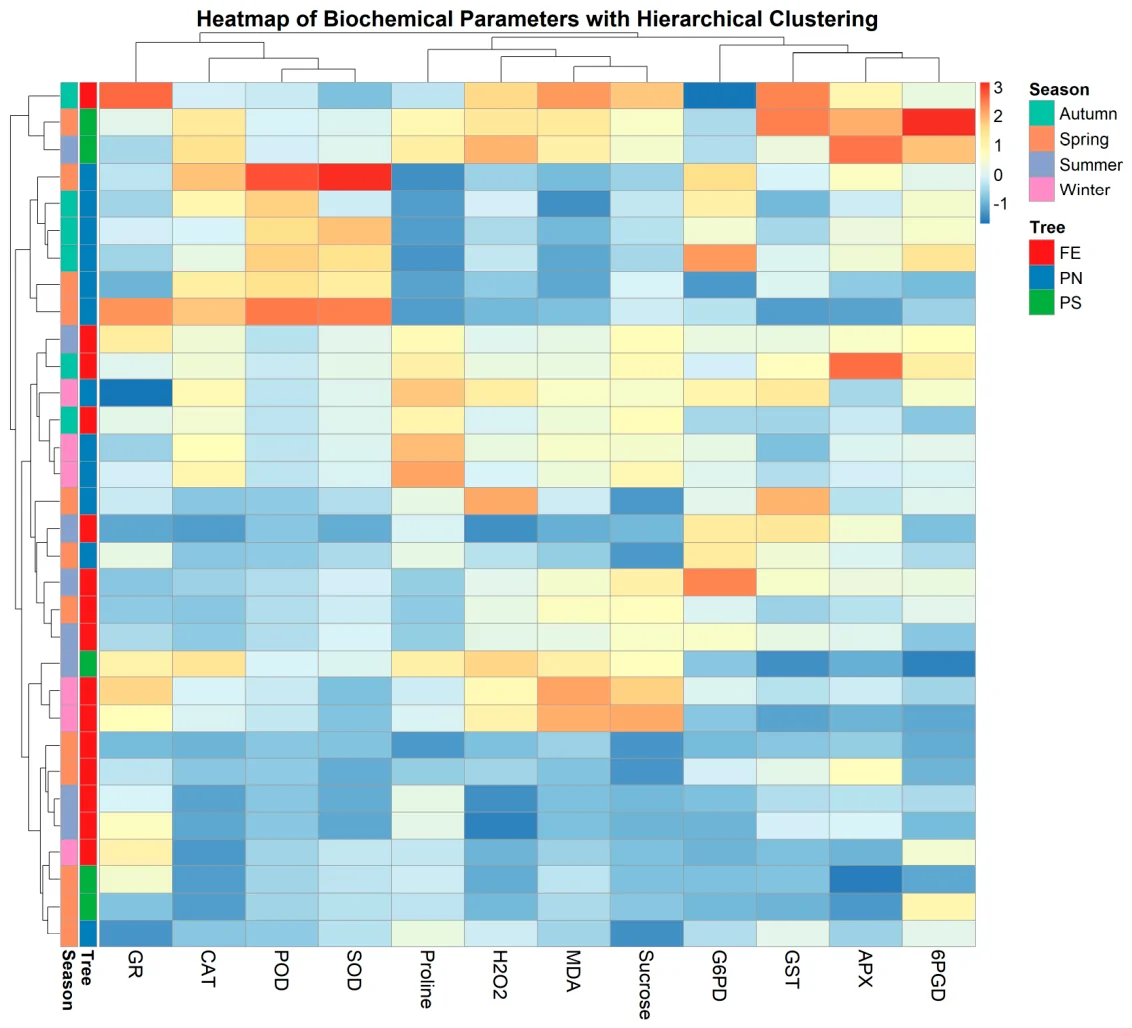
Heatmap of standardized biochemical parameters (z-scores) with hierarchical clustering of samples and traits, annotated by sampling season and focal tree identity.

Hierarchical clustering and heatmap analysis of the biochemical parameters reveal that the variables are structured into three main functional modules (Figure 8): (1) the GR-CAT-POD-SOD block representing enzymatic defense, (2) the Proline-H_2_O_2_-MDA-Sucrose block associated with stress accumulation and metabolic response, and (3) the G6PD-GST-APX-6PGD block governing redox metabolism. Examination of the intensity distribution across the heatmap indicates that high activity values within the enzymatic defense module are predominantly concentrated in samples from the spring and autumn periods. Higher activity values within the enzymatic-defense module were observed more frequently in observations from the focal *P. nigra* individual, whereas relatively high values within the G6PD–GST–APX–6PGD module occurred across observations from the focal *P. sylvestris* and *F. excelsior* individuals. Overall, the clustering illustrates coordinated covariation among biochemical traits within the present dataset rather than evidence of species-specific defense strategies.

## 3. Discussion

The findings of this study indicate that xylem-sap biochemical variation within the three focal trees was associated with focal tree identity, sampling period, and meteorological conditions. The OSI and ADI provide complementary summaries of oxidative-stress- and antioxidant-related variation and support the potential of xylem sap as an integrative matrix for physiological monitoring [5,6,8,10]. The positive associations observed among several oxidative-stress and antioxidant variables suggest related biochemical variation within the dataset. Importantly, the strong H_2_O_2_–MDA association persisted after adjustment for focal tree identity and season, indicating that this relationship was not solely attributable to differences among the three focal trees or seasonal grouping. This association is biologically consistent with the established relationship between ROS accumulation, oxidative membrane damage, and lipid peroxidation under stress conditions [4,28,29]. Previous studies have also shown that the molecular composition of xylem sap can vary in response to biotic and abiotic stress, supporting its potential as a matrix for physiological monitoring [10,30]. Nevertheless, the correlations observed in the present study describe statistical covariation and should not be interpreted as evidence of direct causal coupling. Similarly, the associations of sucrose and proline with oxidative-damage markers should be interpreted cautiously. Proline has well-established roles in plant stress responses, while ROS production and antioxidant regulation are central components of plant responses to environmental stress [31,32]; however, the present associations do not demonstrate that osmotic adjustment and antioxidant defense mechanisms were simultaneously activated. In particular, although MDA and sucrose were strongly correlated in the pooled analysis, this association should not be interpreted as direct evidence that sugar metabolism contributed to stress adaptation. The observed relationship may also reflect seasonal variation in carbon allocation and phenology, differences in sap concentration, or other characteristics of the focal trees. Although soluble sugars are known to function as carbon reserves, signaling molecules, and osmoprotectants under stress conditions [33,34], the mechanism underlying the MDA–sucrose association cannot be resolved from the present dataset.

Principal component analysis revealed that the first major gradient of variation was dominated by oxidative stress indicators and osmolytes, whereas the second gradient reflected antioxidant enzyme activity. This consistent multivariate pattern suggests that the processes of stress perception (ROS accumulation) and stress mitigation (antioxidant activation) represent partially independent physiological dimensions in forest trees [35,36]. Similar findings have been reported across different conifer and broadleaf species, including spruce, pine, oak, and European beech, indicating that this physiological decoupling may be a common feature of tree responses to environmental stress [36,37,38,39]. The distinct clustering of SOD, CAT, and POD within the PCA space is consistent with covariation among these antioxidant enzymes in the present dataset, but does not by itself demonstrate functional synergy. This pattern is nevertheless biologically compatible with their established complementary roles in ROS detoxification: SOD converts superoxide radicals to H_2_O_2_, which can subsequently be detoxified through catalase- and peroxidase-mediated reactions [2,28,40].

Within the present dataset, the focal *P. nigra* individual showed a distinct biochemical profile, being associated in both PCA and RDA with relatively higher POD, SOD, CAT, and G6PD activities than the other two focal trees. This pattern is consistent with a stronger representation of antioxidant and redox-related enzymatic activity in the monitored *P. nigra* individual and is also compatible with previous reports showing responsive antioxidant systems in *Populus* under environmental stress [41]. Elevated G6PD activity is particularly noteworthy because this enzyme contributes to NADPH generation required for glutathione recycling and the maintenance of cellular redox homeostasis [42,43]. The observed association between G6PD and antioxidant enzymes is therefore biologically compatible with an NADPH-linked antioxidant response, although the present data do not demonstrate a direct causal or integrated defense mechanism. Importantly, the present observational design does not establish that the higher enzyme activities directly caused lower oxidative damage. Given the one-individual-per-species design, unbalanced seasonal representation, and differences in tree size, these patterns should be interpreted as characteristics of the monitored focal tree rather than as definitive species-level adaptive traits.

In contrast, the focal *F. excelsior* individual was more closely associated with MDA, sucrose, and GR, reflecting a different biochemical pattern within the present dataset. The elevated MDA concentrations observed in several autumn and winter samples indicate that oxidative damage may accumulate under less favorable environmental conditions. Increased GR activity in the same focal individual may represent a compensatory response aimed at maintaining glutathione redox balance and sustaining antioxidant capacity. Previous studies have demonstrated that GR plays a pivotal role in maintaining cellular redox homeostasis by restoring reduced glutathione pools and is commonly upregulated under conditions of enhanced ROS production [2,44]. The concurrent association of sucrose with MDA and GR is particularly noteworthy, as soluble sugars are known to function not only as osmoprotectants but also as signaling molecules involved in stress acclimation [34,45]. Although the underlying mechanisms were not directly examined in the present study, the clustering of sucrose and GR may indicate a coordinated interaction between carbon metabolism and antioxidant defense under elevated oxidative pressure. Taken together, these associations suggest the co-occurrence of osmotic/metabolic and glutathione-related responses in the focal *F. excelsior* individual within this dataset, rather than demonstrating a species-level adaptive strategy.

The constrained ordination analyses further indicated that TP and WD were the meteorological variables retained after multicollinearity screening and forward selection. Their associations with biochemical variation suggest that meteorological conditions contributed to the observed within-dataset patterns, although the observational design does not establish direct physiological effects. Precipitation is widely recognized as a primary determinant of plant water status and carbon allocation, influencing stomatal conductance, photosynthesis, ROS generation, and antioxidant activity [46,47]. The association observed between TP and MDA, sucrose, and GR indicates that fluctuations in water availability may simultaneously influence membrane stability, osmotic regulation, and glutathione-dependent redox metabolism. Such associations are consistent with the central role of water availability in shaping oxidative stress and antioxidant defense responses in plants.

The significance of WD as a predictor was particularly noteworthy. In the RDA ordination, WD was aligned most closely with POD and, to a lesser extent, with G6PD and SOD, suggesting that wind-related atmospheric variability may be associated with antioxidant defense processes. This interpretation is supported by experimental studies demonstrating that non-injurious wind exposure and other forms of mechanical perturbation can induce peroxidase activity in plants [48,49]. Increased POD activity has been linked to plant defense responses and cell wall reinforcement under mechanical stress, indicating that wind-related exposure may represent a biologically relevant component of the microenvironment rather than merely a physical disturbance. Mechanistically, wind and touch stimuli are known to trigger rapid cytosolic Ca^2+^ transients and associated signaling cascades, which can promote ROS-mediated defense responses [50]. Wind-driven changes in boundary-layer conductance, evaporative demand, and transpiration rates may influence leaf water relations and microclimatic conditions, potentially contributing to physiological pathways linking atmospheric variability with antioxidant responses [51,52]. The weaker alignment of G6PD and SOD with WD may indicate that wind-related atmospheric variability influences broader redox-regulatory processes in addition to peroxidase-mediated defenses. However, because the present analysis is based on correlative relationships, these associations should be interpreted as hypothesis-generating rather than direct evidence of wind-induced enzyme activation.

The Random Forest analyses provided complementary evidence of biochemical differentiation among observations from the three focal trees. CAT emerged as the most important variable for classification, followed by POD and SOD, highlighting the prominent contribution of antioxidant enzymes to the observed multivariate separation. This pattern is consistent with the central role of ROS-detoxification pathways in plant stress physiology, although the present design does not allow these differences to be generalized as species-specific physiological signatures. Catalase is among the most efficient H_2_O_2_-scavenging enzymes and is often considered a key indicator of oxidative stress tolerance because of its high catalytic capacity and low energy cost [2,53]. The dominant contribution of CAT in the Random Forest model is biologically plausible because CAT is responsible for the rapid detoxification of H_2_O_2_, one of the most abundant and potentially damaging reactive oxygen species generated under environmental stress. Accordingly, variation in CAT activity appears to be an important contributor to the biochemical differentiation observed among the three focal trees and may reflect differences in oxidative-stress management, although confirmation at the species level will require independent biological replication. These observations align with recent studies in woody species showing that antioxidant enzyme activity is closely associated with stress resilience [54,55,56], as well as with provenance and species-specific patterns in which higher antioxidant enzyme activities are linked to reduced oxidative damage [57]. In particular, transcriptomic and physiological studies have shown that drought-tolerant clones or provenances maintain elevated POD, SOD, and CAT activities together with enhanced expression of stress-responsive genes under drought conditions [57,58]. Similarly, studies on *Pinus* species have reported coordinated increases in both MDA levels and antioxidant enzyme activities following abiotic stress or biotic attack [59], highlighting the dynamic interplay between oxidative damage and antioxidant defense mechanisms. These lines of evidence corroborate our RDA results, where *P. nigra* aligns with elevated antioxidant activities, whereas *F. excelsior* and parts of the *P. sylvestris* samples align more closely with oxidative-damage and metabolite indicators. Together, the RDA and Random Forest analyses indicate that the biochemical differentiation observed among the three focal trees was associated not only with oxidative-stress-related traits but also with variation in antioxidant regulation and redox-related metabolism. These patterns provide biologically meaningful within-dataset evidence, although their generalization to species-level strategies requires validation using multiple independent individuals per species.

The exploratory Random Forest regression models showed contrasting variable-importance patterns for oxidative stress accumulation and antioxidant defense. For OSI, WD showed the highest importance, followed by TP and focal tree identity, whereas for ADI, TP ranked first, followed by WD and season. These contrasting predictor patterns suggest that oxidative stress accumulation and antioxidant defense may reflect different combinations of meteorological, temporal, and focal-tree-associated variation within the present dataset. The components of OSI (H_2_O_2_, MDA, and proline) are well-established indicators of oxidative stress that respond relatively rapidly to environmental perturbations. H_2_O_2_ functions both as a signaling molecule and an oxidative stress marker, proline accumulates rapidly under osmotic and oxidative stress, whereas MDA reflects membrane lipid peroxidation resulting from ROS-induced cellular damage [31,60,61,62]. These characteristics suggest that OSI is likely to capture relatively short-term physiological responses to changing environmental conditions, explaining its stronger association with dynamic variables such as precipitation and wind direction. The association of wind direction with OSI may reflect atmospheric conditions related to boundary-layer conductance, transpiration, and the canopy microenvironment, potentially influencing plant water relations rather than representing a direct physiological effect of wind itself.

In contrast, ADI reflects the combined activities of several antioxidant enzymes and therefore largely represents the potential capacity of the cellular antioxidant system rather than instantaneous antioxidant activity in vivo. Although antioxidant enzymes can be induced following oxidative stress, their overall capacity is influenced not only by immediate environmental conditions but also by constitutive species-specific expression patterns and longer-term physiological acclimation [63]. These observations indicate that antioxidant defenses are progressively reconfigured throughout seasonal development rather than responding exclusively to short-lived environmental fluctuations. This temporal distinction is consistent with the stronger contribution of season to ADI in the Random Forest models. Seasonal acclimation of antioxidant systems has been widely documented in woody plants, where progressive changes in temperature, photoperiod, and water availability modify antioxidant enzyme activities during annual growth cycles [64,65,66]. Such gradual adjustment of antioxidant capacity contributes to the maintenance of cellular redox homeostasis under fluctuating environmental conditions [62,63]. Therefore, the different predictor structures identified for OSI and ADI likely reflect the distinct biological timescales governing oxidative stress accumulation and antioxidant defense rather than contradictory physiological processes.

While this study provides integrative insights into xylem-sap redox metabolism, some limitations should be acknowledged. First, the experimental design included one focal individual per species, with each individual repeatedly sampled over time. Consequently, tree identity and species identity were fully confounded, and the repeated observations cannot be considered independent biological replicates at the species level. In addition, the numbers of repeated observations were unequal among the focal trees (n = 15 for *F. excelsior*, n = 12 for *P. nigra*, and n = 5 for *P. sylvestris*) because natural sap availability and sampling success varied among sampling periods. Therefore, the biochemical differences observed among the three focal trees should be interpreted as tree-identity-associated patterns within this dataset rather than as generalizable species differences. The multivariate and machine-learning analyses should consequently be regarded as exploratory and hypothesis-generating, and future studies should include multiple independent individuals per species to validate species-level patterns.

Second, the monitoring period encompassed only a single annual cycle across 2023–2024. Consequently, the seasonal and meteorological patterns observed in the present dataset cannot be separated from climatic conditions specific to that monitoring period, and interannual variability could not be evaluated. The associations identified between meteorological variables and xylem-sap biochemical traits should therefore be interpreted as within-period patterns rather than as stable climatic relationships. Multi-year monitoring with repeated seasonal coverage will be necessary to determine whether these associations are consistent across years and under contrasting climatic conditions.

Third, the focal trees were planted individual specimens located within a university campus rather than trees growing within a closed natural forest stand. Consequently, their exposure to local microclimatic conditions and the surrounding soil environment may differ from that of trees in natural forest stands, and these site-specific conditions may have contributed to the observed biochemical variation. The findings should therefore be interpreted within the environmental context of the present campus setting, and extrapolation to natural forest stands should be made cautiously. Future studies across natural stands and contrasting site conditions would help evaluate the broader ecological applicability of the observed patterns.

Fourth, xylem sap composition can be influenced not only by biochemical regulation but also by variation in sap flow, transpiration, sampling time, and tree water status. Because the present dataset did not include direct measurements of sap-flow rate, transpiration rate, or tree water status, possible dilution or concentration effects in the sap cannot be separated from changes in biochemical concentrations or enzyme activities. Therefore, the observed variation in xylem-sap biochemical traits should be interpreted as variation in the measured sap composition rather than as direct evidence of changes in metabolic regulation. Future studies combining biochemical measurements with concurrent sap-flow, transpiration, and water-status measurements would help distinguish metabolic responses from hydrodynamic concentration effects.

Fifth, the use of composite indices (OSI and ADI) and multivariate models such as Random Forest provides a complementary exploratory assessment of environmental associations, but these relationships should be interpreted as hypothesis-generating rather than definitive evidence of causality. Nevertheless, integrating multiple analytical approaches provides complementary perspectives on the observed patterns. Future research with higher temporal resolution and broader biological replication across diverse microhabitats will be necessary to evaluate their generalizability. Despite these constraints, the present study provides an exploratory baseline for examining associations between environmental variability and xylem-sap biochemical patterns in repeatedly monitored focal trees.

## 4. Materials and Methods

### 4.1. Study Area and Plant Material

This study included three focal tree individuals, each representing a different species: Scots pine (*P. sylvestris* L.), European ash (*F. excelsior* L.), and black poplar (*P. nigra* L.). Sap samples were obtained from standing trees during their natural flow periods and evaluated according to different sampling periods.

To ensure regular and uninterrupted sampling, the study material was selected within the Ağdacı Campus of Bartın University. The site has a northwest-facing slope; the trees used in the study do not form a closed stand but consist of individual specimens established through planting within the campus.

The geographic coordinates and elevations of the sampled trees are: 41.600637° N, 32.346190° E (66 m) for *P. sylvestris*; 41.600569° N, 32.346128° E (65 m) for *F. excelsior*; and 41.600421° N, 32.346719° E (70 m) for *P. nigra*. Height and diameter characteristics were measured prior to sampling. *P. sylvestris* was approximately 10 m tall with an 18 cm diameter at breast height (DBH), *F. excelsior* was ~10 m tall with 16 cm DBH, and *P. nigra* was ~18 m tall with 30 cm DBH.

Sampling was conducted between 2023 and 2024. The study followed three focal tree individuals, with one individual representing each species. The same individuals were repeatedly sampled over the monitoring period; therefore, the 32 observations represent repeated temporal measurements rather than independent biological replicates. A total of 15 observations were obtained from the focal *F. excelsior* individual, 12 from the focal *P. nigra* individual, and 5 from the focal *P. sylvestris* individual. Samples were classified into spring, summer, autumn, and winter periods. Seasonal representation was unbalanced across the three focal trees: the focal *F. excelsior* individual was represented in spring (n = 3), summer (n = 6), autumn (n = 3), and winter (n = 3); the focal *P. nigra* individual in spring (n = 6), autumn (n = 3), and winter (n = 3); and the focal *P. sylvestris* individual in spring (n = 3) and summer (n = 2). Consequently, focal tree identity and season were not fully crossed in the sampling design, and their effects could not be completely disentangled. Differences in natural sap flow and sampling success among sampling periods resulted in unequal numbers of repeated observations across the three focal trees. Because each species was represented by a single focal individual, tree identity and species identity were fully confounded in the present design, and species-level effects could not be separated from individual-tree effects.

### 4.2. Xylem Sap Collection

Xylem sap was collected from each focal tree using a custom-built stem-tapping apparatus during periods of natural sap flow. At each sampling event, an 8-mm-diameter borehole was drilled approximately 20–25 cm above ground level and extended approximately 2–3 cm into the sapwood at a slight angle to facilitate passive sap flow. The first emerging sap fraction was discarded to minimize potential contamination from cellular debris, disrupted tissues, and oxidized material generated during drilling. Only the subsequently exuding sap was used for biochemical analyses; no bark, wood tissue, or drilling debris was included in the collected samples. Because sap was obtained by passive flow rather than by tissue homogenization or mechanical extraction of the sampled wood, this procedure was intended to minimize the contribution of cellular contents released from disrupted xylem-associated tissues. However, no independent cellular-contamination marker was measured, and complete exclusion of minor contamination from damaged cells cannot be demonstrated. Subsequently, sap was collected into 2-mL Eppendorf tubes wrapped in aluminum foil to minimize light exposure. Collection was conducted at variable times during daylight hours depending on natural sap flow and field conditions, and individual tubes were replaced once sufficient sap had accumulated. Immediately after collection, samples were transported to the laboratory on ice and stored at 4 °C until biochemical analysis. For successive sampling events, a new borehole was opened approximately 5–10 cm lateral to or above the previous sampling point. After sampling, each borehole was sealed to reduce the risk of water entry and pathogen infection. The same general passive stem-tapping procedure was applied to all three focal trees, and no species-specific extraction method was used for *P. sylvestris*. For the focal *P. sylvestris* individual, the same initial-fraction discard procedure was applied before collection of the subsequently exuding sap; no additional species-specific extraction procedure was used. Natural sap availability and sampling success varied among sampling events; however, sap yield was not quantified as a separate response variable and was therefore not statistically compared among the focal trees.

### 4.3. Biochemical and Meteorological Variables

The meteorological variables used in the analyses included average wind speed (AWS), wind direction (WD), total precipitation (TP), relative humidity (RH), air temperature (T), solar radiation (SR), sunshine duration (SD), soil temperature at 50 cm (ST50) and at 100 cm (ST100). Meteorological data were obtained from the Bartın University Weather Station located within the study area, ensuring spatial correspondence between environmental measurements and sap sampling locations. Meteorological variables were matched to each xylem-sap sampling event using the daily records corresponding to the sampling date. Daily mean values were used for the meteorological variables, whereas precipitation was represented by the total precipitation recorded for the corresponding day.

Biochemical assessments employed parameters representing oxidative stress, osmotic adjustment, antioxidant defense and redox metabolism. Oxidative stress markers comprised H_2_O_2_ and MDA. Osmotic adjustment was represented by proline and sucrose contents. Antioxidant defense was evaluated by activities of CAT, POD, SOD and APX. Redox metabolism was assessed using activities of GR, GST, G6PD and 6PGD.

H_2_O_2_ was determined spectrophotometrically at 390 nm according to Loreto and Velikova [67], while MDA was determined according to Heath and Packer [68] using spectrophotometric measurements based on the 532/600 nm absorbance difference. Proline content was determined at 528 nm following Bates et al. [69], and sucrose content was measured at 620 nm according to Wu et al. [70]. CAT, POD, SOD, and APX activities were determined spectrophotometrically according to Sairam and Srivastava [71]. GR and GST activities were determined according to Ozturk et al. [72]. G6PD and 6PGD activities were determined spectrophotometrically at 340 nm following Beutler [73]. For enzymatic analyses, 1% (*w*/*v*) polyvinylpyrrolidone (PVP) was included in the extraction buffer to minimize interference from phenolic compounds and support enzyme stability. Enzyme extraction procedures were performed on ice and at 4 °C to minimize proteolytic activity and preserve enzyme activity during sample processing. All biochemical measurements were performed in technical triplicate. H_2_O_2_, MDA, and proline contents were expressed as mmol kg^−1^ xylem sap, sucrose as % (*w*/*v*), and CAT, POD, SOD, APX, GR, GST, G6PD, and 6PGD activities as U g^−1^ xylem sap.

To summarize overall plant physiological status, standardized biochemical variables were used to calculate the OSI and ADI, which were then employed in the machine learning analyses.

### 4.4. Statistical and Multivariate Analyses

Pearson correlation analysis was performed separately for meteorological variables and biochemical parameters to identify pairwise relationships and potential collinearity patterns. Because pooled biochemical correlations could partly reflect differences among the three focal tree identities or seasonal sampling structure, adjusted correlations were additionally calculated after removing the effects of focal tree identity and season. For each biochemical variable, residuals were obtained from a linear model including focal tree identity and season as categorical predictors, and Pearson correlations among these residuals were used to quantify biochemical associations after adjustment for these grouping factors. To reduce multicollinearity among meteorological predictors, Variance inflation factors (VIFs) were calculated iteratively from coefficients of determination obtained from ordinary least-squares models, and predictors with VIF values >10 were sequentially removed.

A forward-selection procedure based on permutation tests was then applied to identify the meteorological variables that significantly explained variation in biochemical responses. Principal Component Analysis (PCA) was performed using centered and standardized biochemical data to visualize major gradients of variation among samples. Because each species was represented by a single repeatedly sampled individual, tree identity was treated as a descriptive grouping factor rather than as a replicated species-level experimental factor. Accordingly, multivariate analyses involving this grouping were interpreted as exploratory comparisons among the three focal individuals and not as tests of generalizable species effects. An exploratory PERMANOVA was used to assess multivariate differentiation among observations associated with the three focal tree identities, based on Euclidean distance matrices with 999 permutations. Because the numbers of repeated observations were unequal among the focal trees, homogeneity of multivariate dispersion was additionally assessed using PERMDISP (distance to group centroids) with 999 permutations. The PERMDISP test did not indicate a statistically significant difference in multivariate dispersion among the three focal trees (F = 2.6545, *p* = 0.084). Redundancy Analysis (RDA) was subsequently employed to examine associations between focal tree identity, selected meteorological variables, and the overall biochemical profile. The significance of constrained models was evaluated using permutation-based testing. Exploratory variation partitioning was used to summarize the variation associated with focal tree identity, season, and meteorological variables using adjusted R^2^ values. Because seasonal coverage was incomplete across focal trees, the resulting unique and shared fractions were interpreted descriptively rather than as a complete separation of independent tree and seasonal effects.

Random Forest models were used for two exploratory purposes. First, a classification model was fitted to identify the biochemical variables contributing most strongly to the classification of observations by focal tree identity. The model included the measured biochemical variables as predictors and focal tree identity as the response, and was fitted to complete-case observations using the randomForest package (version 4.7.1.2) with 1000 trees. Variable importance was evaluated using Mean Decrease Accuracy and Mean Decrease Gini. Second, separate Random Forest regression models were fitted for the Oxidative Stress Index (OSI) and Antioxidant Defense Index (ADI) using the ranger package (version 0.18.0) with 1000 trees. Focal tree identity, season, and the meteorological variables retained after the preceding multicollinearity screening and forward-selection procedure were included as predictors, and predictor importance was quantified using permutation importance. A fixed random seed (set.seed = 42) was used to ensure reproducibility. Because of the limited sample size and the one-individual-per-species design, these Random Forest models were used for exploratory variable-importance ranking rather than for validated predictive inference or generalizable species classification.

To summarize the coordinated damage and defense responses for these regression models, the two composite indices (OSI and ADI) were developed. Given that the individual biochemical parameters were measured in different units and scales, each variable (x) was first standardized using z-score transformation $\left(z=(x-\mu )/\sigma \right)$. OSI was then calculated as the arithmetic mean of the standardized values for H_2_O_2_, MDA, and Proline. Similarly, ADI was calculated as the mean of the standardized values for the antioxidant enzymes (CAT, POD, SOD, APX, GR, GST, G6PD, and 6PGD).

Finally, hierarchical clustering analysis was performed using Euclidean distance and complete-linkage clustering. Results were visualized as a heatmap after z-score standardization of biochemical variables to enable the identification of coordinated response patterns across focal tree identities and sampling seasons. All analyses were conducted using R software (version 4.5.1). The vegan package (version 2.7.3) was used for PERMANOVA, RDA, forward selection, permutation-based significance testing, PERMDISP, and variation partitioning. Random Forest classification was implemented using the randomForest package (version 4.7.1.2), whereas regression models for OSI and ADI were fitted using the ranger package (version 0.18.0). Correlation matrices were generated using the corrplot package (version 0.95), hierarchical clustering and heatmaps using the pheatmap package (version 1.0.13), and graphical outputs were primarily produced using the ggplot2 package (version 4.0.2) and the ggrepel package (version 0.9.8).

## 5. Conclusions

Within the three repeatedly sampled focal trees, xylem sap biochemistry varied in association with tree identity, sampling period, and meteorological conditions. The observed associations among oxidative stress markers, osmolytes, and antioxidant enzymes suggest that xylem sap functions as an integrative matrix reflecting both stress perception and defense regulation rather than a passive transport fluid. In particular, the focal *P. nigra* individual showed a more pronounced enzymatic defense profile, whereas the focal *F. excelsior* and *P. sylvestris* individuals were more closely associated with oxidative-damage and metabolic-adjustment traits. The multivariate and machine learning analyses provided complementary perspectives on these patterns. PCA, PERMANOVA, and RDA revealed multivariate structuring among observations associated with the three focal tree identities, while exploratory Random Forest models highlighted catalase and selected meteorological variables as important contributors to focal-tree classification and variation in the composite stress indices. Variation partitioning further indicated that meteorological variables accounted for a notable fraction of the biochemical variation within the present dataset, alongside variation associated with focal tree identity and season. Taken together, these findings support the potential of xylem sap as a complementary indicator of tree physiological responses and its further evaluation in ecophysiological monitoring studies under changing environmental conditions.

From a methodological perspective, the integration of multivariate ordination and machine-learning approaches provided a useful exploratory framework for examining the complex variation in xylem sap biochemistry and may support future ecophysiological monitoring studies. These findings suggest that xylem sap, particularly when evaluated together with composite indices such as OSI and ADI, has potential as a complementary matrix for monitoring physiological responses to environmental variability in trees. Future studies should incorporate higher-resolution temporal sampling and include additional edaphic and biotic factors to further examine associations between environmental variability and xylem-sap redox-related biochemistry.

## Figures and Tables

**Table 1:** Exploratory PERMANOVA results for multivariate differentiation among observations grouped by focal tree identity.

Source	df	Sum of Squares	R^2^	F	*p*-Value
Focal tree identity	2	52.3	0.1406	2.372	0.008
Residual	29	319.7	0.8594	–	–
Total	31	372	1	–	–

**Table 2:** VIF screening and forward selection results for meteorological variables.

Variable	Initial VIF	Retained After VIF Screening	Selected by Forward Selection
ST50	376.05	No	–
ST100	305.05	Yes	No
SR	88.27	Yes	No
SD	84.22	No	–
T	29.7	No	–
RH	13.27	Yes	No
TP	3.96	Yes	Yes
AWS	3.59	Yes	No
WD	2.5	Yes	Yes

Initial VIF values are reported. Iterative VIF screening sequentially removed ST50, SD and T, yielding a final predictor set consisting of AWS, WD, TP, RH, SR and ST100. Forward selection based on adjusted R^2^ retained only total precipitation (TP) and wind direction (WD) as significant explanatory variables.

## Data Availability

The individual biochemical observations supporting the findings of this study are provided in Supplementary Table S1. Additional data are available from the corresponding author on reasonable request.

## Supplementary Materials

The following supporting information can be downloaded at: https://www.mdpi.com/article/10.3390/plants15172549/s1, Table S1: Individual xylem-sap biochemical observations obtained from the three repeatedly sampled focal trees across sampling seasons; Figure S1: Adjusted relationships between H_2_O_2_ and MDA and between POD and SOD after controlling for focal tree identity and season.

## Abbreviations

The following abbreviations are used in this manuscript:

6PGD	6-Phosphogluconate dehydrogenase
ADI	Antioxidant Defense Index
APX	Ascorbate peroxidase
AWS	Average wind speed
CAT	Catalase
DBH	Diameter at breast height
G6PD	Glucose-6-phosphate dehydrogenase
GR	Glutathione reductase
GST	Glutathione S-transferase
H_2_O_2_	Hydrogen peroxide
MDA	Malondialdehyde
NADPH	Nicotinamide adenine dinucleotide phosphate
OSI	Oxidative Stress Index
PCA	Principal Component Analysis
PERMANOVA	Permutational Multivariate Analysis of Variance
POD	Peroxidase
RDA	Redundancy Analysis
RF	Random Forest
RH	Relative humidity
ROS	Reactive oxygen species
SD	Sunshine duration
SOD	Superoxide dismutase
SR	Solar radiation
ST50	Soil temperature at 50 cm depth
ST100	Soil temperature at 100 cm depth
T	Air temperature
TP	Total precipitation
VIF	Variance Inflation Factor
WD	Wind direction
