# Supplementary material for: Integrating Multivariate Ordination and Machine Learning to Disentangle the Environmental Drivers of Xylem Sap Redox Metabolism in Trees
Source: Plants (Basel). 2026 Aug 22;15(17):2549. doi: 10.3390/plants15172549 (PMC13567610; doi:10.3390/plants15172549)
Supplement: Supplementary file 1 [file plants-15-02549-s001.zip › Table S1. Individual xylem-sap biochemical observations obtained from the three repeatedly sampled focal trees across sampling seasons.pdf]

Table S1. Individual xylem-sap biochemical observations obtained from the three repeatedly sampled focal trees across sampling seasons.

Tree	Season	Oxidative Stress Markers		Osmolytes/Metabolites		Antioxidant Enzymes				Antioxidant and Redox Metabolism Enzymes			
		H_2O_2	MDA	Proline	Sucrose	CAT	POD	SOD	APX	GR	GST	G6PD	6PGD
FE	Spring	29.35	26.94	0.25	0.97	160.50	1043.65	266.30	160.90	321.64	185.99	345.56	386.45
FE	Spring	33.86	22.28	0.59	0.97	207.13	1430.75	123.44	238.58	412.61	245.93	402.82	394.69
FE	Spring	46.51	59.33	0.54	6.56	201.68	3880.01	584.67	176.34	354.91	196.41	414.96	520.40
FE	Summer	45.77	48.22	0.57	6.26	218.97	3959.72	637.98	202.64	391.31	248.21	461.48	425.00
FE	Summer	45.28	56.12	0.57	7.62	228.57	3822.39	611.33	216.50	350.25	271.48	601.49	536.23
FE	Summer	20.04	15.90	0.91	2.00	113.63	810.31	122.76	225.11	289.56	321.11	511.81	414.30
FE	Summer	19.76	19.87	1.04	2.00	124.76	1006.11	138.54	176.87	448.09	208.03	346.70	454.32
FE	Summer	18.01	19.82	1.01	1.93	133.27	968.87	106.98	193.43	557.79	226.77	337.27	405.90
FE	Summer	44.46	48.07	1.38	6.78	420.87	4339.87	736.46	233.35	640.33	252.97	433.91	591.31
FE	Autumn	43.02	51.93	1.45	6.87	430.05	4797.53	715.42	184.13	486.29	198.78	373.61	422.93
FE	Autumn	47.62	49.87	1.51	7.01	419.32	5213.11	757.50	348.20	473.66	281.46	404.34	629.92
FE	Autumn	69.81	99.82	0.77	9.43	323.86	5292.69	256.62	251.07	858.12	393.18	281.35	536.93
FE	Winter	57.30	96.96	0.84	9.18	327.84	5453.07	255.33	187.41	707.35	210.00	415.44	443.41
FE	Winter	60.50	94.71	0.92	10.25	337.49	5116.90	263.03	139.09	575.75	155.56	354.64	378.52
FE	Winter	26.40	26.70	0.78	2.31	110.88	2539.74	540.17	139.03	619.67	177.92	334.60	559.57
PS	Spring	25.16	33.99	0.85	2.35	117.96	2500.23	507.44	106.16	534.63	180.82	348.97	379.76
PS	Spring	27.57	30.00	0.76	2.61	120.91	2592.83	491.07	122.44	338.36	170.31	338.61	610.91
PS	Spring	65.32	75.53	1.40	6.26	576.52	6457.54	676.05	314.98	480.05	395.19	376.34	854.78
PS	Summer	76.34	70.64	1.53	6.10	616.71	6329.45	700.96	344.59	380.00	255.43	380.89	706.00
PS	Summer	70.38	71.37	1.49	6.50	591.00	6822.61	684.59	136.06	614.85	145.29	354.15	335.02
PN	Spring	77.74	37.54	1.03	1.15	203.92	1174.59	475.39	176.28	426.95	362.78	424.76	515.48
PN	Spring	37.27	24.97	1.03	1.10	204.74	1172.96	452.76	199.12	495.88	263.26	511.73	457.36
PN	Spring	40.04	27.40	1.06	0.92	203.92	1166.43	498.03	163.08	258.93	241.36	382.51	520.90
PN	Winter	62.49	57.74	1.86	6.12	495.29	4561.28	702.97	168.50	208.48	318.65	491.93	567.40
PN	Winter	47.46	57.51	1.92	6.05	483.68	4717.92	679.38	197.84	366.23	178.78	431.95	521.71
PN	Winter	41.85	51.98	2.03	7.12	519.09	4684.89	660.51	191.46	443.66	206.57	418.02	504.88
PN	Autumn	40.94	6.10	0.27	3.90	514.29	21223.34	590.43	186.96	373.53	171.97	498.69	560.07
PN	Autumn	35.27	17.42	0.26	3.66	330.22	19962.39	1579.77	214.85	438.81	202.56	449.71	566.56
PN	Autumn	38.79	13.31	0.22	3.29	379.14	21229.15	1417.81	221.81	374.72	235.57	586.36	658.53
PN	Spring	31.36	13.39	0.30	4.42	553.67	19575.53	1269.52	155.84	311.79	237.86	314.30	407.12
PN	Spring	27.11	20.63	0.26	4.15	664.40	27990.05	1889.44	127.94	800.71	154.96	386.77	439.72
PN	Spring	33.25	19.54	0.20	2.99	672.63	31043.44	2231.49	234.45	411.17	229.32	533.30	519.06
